# Fast-Track Extubation After Cardiac Surgery: A Narrative Review

**DOI:** 10.3390/jcdd13010006

**Published:** 2025-12-22

**Authors:** Alexa Christophides, Stephen DiMaria, Sophia Ann Jacob, Andrew Feit, Jonathan Oster, Sergio Bergese

**Affiliations:** 1 Office of the Vice President of Medical Affairs, Northwell Mather Hospital, Port Jefferson, NY 11777, USA; achristophides@northwell.edu; 2 Department of Anesthesiology, Stony Brook University Hospital, Stony Brook, NY 11794, USA; stefano.dimaria@stonybrookmedicine.edu (S.D.); jonathan.oster@stonybrookmedicine.edu (J.O.)

**Keywords:** early extubation, multidisciplinary protocol, intensive care unit length of stay, resource utilization, postoperative recovery, enhanced recovery after surgery, hemodynamic stability, patient selection, anesthesia management, reintubation

## Abstract

Fast-track extubation has emerged as a vital component of Enhanced Recovery After Surgery pathways, designed to optimize recovery and resource utilization after cardiac surgery, contrasting with traditional prolonged ventilation. This review explores the evidence supporting fast-track extubation, detailing patient selection criteria based on preoperative risk factors and functional status and outlining perioperative management strategies. It synthesizes findings from various studies, including randomized controlled trials, retrospective studies, and meta-analyses, focusing on intraoperative techniques such as low-dose opioids, neuromuscular blockade reversal, controlled cardiopulmonary bypass duration, judicious inotrope use, and minimal transfusion, alongside structured postoperative protocols emphasizing early sedative weaning and spontaneous breathing trials. Results demonstrate that fast-track extubation decreases intensive care unit stay, reduces costs and ventilator-associated complications, with a safety comparable to conventional care. Prolonged cardiopulmonary bypass time, dependency on inotropes, and intraoperative blood transfusions are identified as critical predictors of fast-track extubation failure. In conclusion, the successful implementation of fast-track extubation protocols requires a collaborative, multidisciplinary approach, proving essential for improving patient outcomes, minimizing complications such as postoperative delirium, and enhancing hospital efficiency in cardiac surgery. Further research should aim to refine patient selection and standardize protocols across healthcare systems.

## 1. Introduction

Enhanced recovery after surgery (ERAS) has become a primary focus in modern perioperative care, emphasizing optimization of patient outcomes while decreasing hospital length of stay (HLOS) through multidisciplinary, patient-centered, evidence-based pathways [1]. Rooted in principles of physiologic optimization, early mobilization, and reduction in perioperative stress responses, ERAS has reshaped perioperative workflows and created new expectations for postoperative recovery across surgical specialties. Within this framework, FTE has emerged as a central strategy to support earlier mobilization, improved resource utilization, and enhanced postoperative recovery in cardiac surgical patients [2,3,4].

Traditionally, prolonged postoperative mechanical ventilation following cardiac surgery was considered advantageous, providing a controlled environment for hemodynamic stabilization and gas exchange while minimizing myocardial oxygen demand [5]. This conservative approach reflected earlier eras of cardiac surgery when anesthetic techniques, cardiopulmonary bypass management, and postoperative monitoring capabilities were more limited. However, advances in surgical precision, myocardial protection strategies, perioperative analgesia, and hemodynamic monitoring have enabled the safe implementation of extubation protocols that support earlier liberation from mechanical ventilation with demonstrated improvements in recovery [6,7].

Successful fast-track extubation (FTE) requires careful patient selection, intraoperative management, and structured postoperative protocols. Preoperative functional status, comorbidities, anesthetic approaches, adequacy of neuromuscular blockade reversal, and hemodynamic stability are all critical factors in determining readiness for extubation [8,9]. Early identification of appropriate candidates can mitigate perioperative risk and reduce the likelihood of extubation failure. Equally important is coordinated involvement of anesthesiologists, surgeons, intensivists, nurses, and respiratory therapists, as multidisciplinary collaboration has consistently been shown to enhance safety, reduce variability, and strengthen protocol adherence.

Evidence consistently demonstrates that FTE reduces intensive care unit (ICU) stay, HLOS, ventilator-associated pneumonia, and healthcare costs while maintaining safety comparable to conventional care [10]. These benefits extend beyond clinical outcomes; earlier mobilization and shorter ventilation duration also translate into improved patient satisfaction and more efficient use of critical care resources. Despite these advantages, challenges persist in standardizing FTE protocols across institutions, as patient populations, available resources, and provider expertise vary widely [2]. Such differences influence feasibility, extubation timelines, and success rates, highlighting the need for adaptable, evidence-based frameworks.

This review explores the evidence supporting FTE, examining patient selection, perioperative management, and institutional protocols that facilitate safe and effective early extubation. By addressing both benefits and limitations, the aim is to clarify FTE’s role in modern cardiac surgical recovery and highlight its potential to improve outcomes, minimize complications and enhance hospital efficiency. Through a synthesis of contemporary data and practical considerations, this review defines the evolving role of FTE within ERAS-based cardiac care and identifies opportunities for further refinement and research (Table S1. Summary of selected fast-track extubation (FTE) studies in adult cardiac surgery).

## 2. Epidemiology of Cardiac Surgery

Cardiovascular disease remains the leading cause of death in the United States [11]. In 2019, 47.5% of adults aged ≥55 reported cardiovascular disease [12]. Cardiac surgery remains central to management, with evolving procedural innovations [13]. Coronary artery bypass grafting (CABG) is the most common adult cardiac operation, performed 161,816 times in 2019, with CABG combined with aortic valve replacement performed 14,246 times [14,15]. Isolated aortic valve replacement (20,965 cases), isolated mitral valve repair (12,570), mitral valve replacement (10,748), and mitral repair with CABG (2624) represent other common procedures [15]. Transcatheter aortic valve replacement (TAVR), first introduced in 2002, exceeded all surgical aortic valve replacements by 2019 (72,991 vs. 57,626) [15,16]. Between 2015 and 2018, 23,777 congenital heart surgeries were reported, and heart transplantation reached a record 3817 procedures in 2021 [15,17].

Given operative and perioperative risks, surgical decisions are made by a multidisciplinary “heart team,” typically including cardiothoracic surgeons, cardiologists, and anesthesiologists [18]. Preoperative imaging with echocardiography, CT, or MRI guides case selection and surgical planning [18].

CABG remains the standard for severe coronary artery disease. According to 2021 ACCF/AHA guidelines, CABG improves survival in stable ischemic heart disease with multivessel disease and LVEF < 35% or significant left main stenosis (class I recommendation) [19]. Patients undergoing CABG are typically older (~65 years), male, and of lower socioeconomic status, often with dyslipidemia, diabetes, and hypertension [20,21,22].

Valvular surgery follows consistent American and European guidelines, with initial evaluation distinguishing stenotic from regurgitant lesions and grading severity [18,23]. Severe aortic stenosis (valve area < 1 cm^2^) warrants replacement in symptomatic patients or those with LV dysfunction [24,25]. High-risk patients may undergo TAVR based on EUROScore or STS criteria, comorbidities, or frailty [26,27,28]. Patients are typically >65 years with hypertension, atrial fibrillation, and coronary disease; younger patients often have congenital abnormalities [29,30,31,32].

Severe aortic regurgitation (vena contracta > 0.6 cm) is treated surgically in symptomatic patients, those with LVEF < 50%, or significant ventricular dilatation [25,33,34]. Acute cases may result from endocarditis or dissection, and chronic disease carries high morbidity and mortality [35,36]. In Western countries, etiology is usually degenerative or congenital, while rheumatic disease predominates in developing nations [34,35].

Severe mitral regurgitation (effective regurgitant orifice > 0.2 cm^2^, regurgitant volume > 30 mL, or fraction > 50%) is surgically indicated in symptomatic patients and in asymptomatic patients with systolic dysfunction [37,38]. Etiology is typically degenerative in the United States and rheumatic in developing countries [25]. Mitral repair is preferred over replacement and is the most common MR procedure in North America [39]. Mitral stenosis is rare but may require intervention; patients are generally 40–75 years old with roughly equal gender distribution and may have concomitant valve disease or pulmonary hypertension [40].

## 3. Enhanced Recovery After Cardiac Surgery

ERAS pathways in cardiac surgery build upon evidence-based, multimodal strategies designed to optimize physiologic function before, during, and after the operative course. These pathways emphasize coordinated interdisciplinary care that reduces variability, streamlines workflow, and support earlier recovery milestones following complex surgical interventions [41]. Core ERAS elements, spanning preoperative optimization, intraoperative standardization, and postoperative rehabilitation, have been repeatedly associated with reductions in postoperative complications, ICU length of stay, and HLOS [42,43,44].

In the preoperative phase, ERAS guidelines emphasize optimization of comorbidities, nutrition, and glycemic control, each of which directly influences postoperative ventilation needs and hemodynamic stability. For example, aggressive perioperative glycemic management has been demonstrated to reduce surgical site infection rates and improve wound healing in cardiac patients [41,42]. Smoking cessation, recommended across all ERAS programs, mitigates respiratory complications and enhances postoperative pulmonary mechanics, reducing the likelihood of prolonged ventilation [45]. Preoperative counseling regarding early extubation expectations additionally prepares patients and improves adherence to rehabilitation goals, reinforcing the patient-centered foundation of ERAS pathways [41].

During the intraoperative period, ERAS protocols promote opioids-sparing technique regimens, targeted hemodynamic management, normothermia, and adherence to goal-directed fluid therapy. These elements improve postoperative respiratory function and stabilize cardiopulmonary physiology, both of which are critical for facilitating early extubation [43,44]. Multimodal analgesia, including regional anesthesia when appropriate, reduces opioid exposure, which in turn lessens respiratory depression and delirium risk, two major contributions to delayed extubation [46,47]. Standardization of anesthetic approaches also reduces inter-provider variability, allowing more reliable prediction of extubation readiness as patients transition from the operating room to the ICU [48,49].

In the postoperative setting, ERAS protocols emphasize early mobilization, effective analgesia, structured sedation weaning, and systematic respiratory optimization. Early mobilization, which has been shown to improve respiratory mechanics and decrease atelectasis, also accelerates the return of functional independence [50,51,52]. Multimodal pain management strategies improve respiratory drive, reduce opioid requirements, and facilitate participation in early mobility and breathing exercises [46,47]. Postoperative delirium, a substantial barrier to FTE, is mitigated through multimodal analgesia, attention to sleep–wake regulation, and the use of light sedative agents such as dexmedetomidine [53].

ERAS cardiac programs have been evaluated across multiple institutions, consistently demonstrating significant improvements in postoperative ventilation time, ICU stay and recovery trajectories. Li et al. reported that ERAS implementation reduced ICU LOS and complication rates in a randomized clinical trial of adult cardiac surgery patients, while Petersen et al. showed substantial economic benefit, including lower hospitalization costs and shorter length of stay, without increasing adverse events [42,43]. A propensity-matched analysis by Yazdchi et al. further confirmed reductions in mechanical ventilation duration, early mobilization time, and 30-day readmission rates following ERAS adoption [44]. Collectively, these findings support the conclusion that ERAS pathways not only standardize high-quality perioperative care, but additionally materially improve outcomes in adult cardiac surgery.

The integration of FTE within ERAS frameworks is particularly synergistic. Early removal of mechanical ventilation promotes patient comfort, decreases ventilator-associated complications, and allows faster progression to ERAS milestones such as mobilization and enteral nutrition [2,3,4,6,10,54].

## 4. Fast-Track Cardiac Anesthesia

Historically, cardiac anesthesia relied on high-dose opioids with postoperative mechanical ventilation overnight in the ICU. In the 1990s, “fast-track cardiac anesthesia” (FTCA) emerged to conserve resources and improve recovery. FTCA generally involves low-dose opioid anesthesia combined with time-directed extubation protocols, aiming for early extubation (within eight hours), early ambulation, and early enteral intake [48]. Limiting opioid-induced respiratory depression facilitates earlier extubation, while standardized protocols reduce inter-institutional variability.

A 2016 Cochrane review comparing low-dose opioid FTCA with conventional high-dose opioid anesthesia found no differences in mortality, reintubation, myocardial infarction, or stroke, demonstrating comparable safety. FTCA reduced ICU stay but did not significantly shorten overall hospitalization [49].

Early extubation enables timely physical therapy, mitigating ICU-acquired weakness and associated complications such as pneumonia, thromboembolism, and pressure ulcers. Although early mobilization improves functional outcomes, optimal protocols and therapy frequency remain undefined, and staff-intensive interventions may increase resource utilization [50].

Early extubation also reduces postoperative delirium, a risk heightened by CPB due to altered cerebral perfusion and ischemic byproducts. Prolonged mechanical ventilation independently increases delirium risk by disrupting sleep–wake cycles and cardiopulmonary physiology; thus, earlier extubation mitigates this risk in vulnerable cardiac surgery patients [55].

FTCA also has economic benefits. Petersen et al. evaluated ERAS protocols for minimally invasive valve surgery, incorporating prehabilitation, intraoperative optimization, and early postoperative mobilization. Patients extubated in the operating room participated in physical therapy within three hours, had lines and drains removed within 12 h, and were discharged on postoperative days four or five. Compared with conventional care, ERAS reduced operating room time, ICU stay, overall hospitalization, and total costs, despite higher physical therapy costs, without increasing complications [43].

Collectively, FTCA and cardiac ERAS protocols demonstrate improved patient outcomes, shorter ICU stays, and economic benefits, supporting their adoption in contemporary cardiac surgery practice.

## 5. Perioperative Management

### 5.1. Patient Selection

Identifying appropriate candidates for FTE after cardiac surgery is critical to ensuring patient safety and optimizing outcomes. Not all patients are ideal candidates, as certain preoperative factors and comorbidities can increase the risk of respiratory complications and reintubation [9,56]. However, recent retrospective data suggest that common comorbidities such as diabetes and hypertension may not significantly affect extubation outcomes, whereas factors including high inotropic support, late-night case timing, and hypothermia are more strongly associated with delayed extubation [3]. Candidate selection requires a comprehensive evaluation of preoperative history, functional status, and comorbidities.

FTE is generally more feasible in patients undergoing less invasive procedures, such as off-pump CABG or minimally invasive valve repair, whereas complex surgeries with anticipated prolonged bypass times carry increased risk [57]. Age alone is not an absolute contraindication; however, elderly and frail patients may have decreased physiological reserve. Comprehensive geriatric assessment can identify elderly patients who remain suitable candidates despite advanced age [58]. Lifestyle factors, including smoking and chronic alcohol use, should be assessed, with preoperative smoking cessation recommended to improve postoperative respiratory outcomes [45].

Assessment of pulmonary status is particularly important in patients with chronic obstructive pulmonary disease (COPD) or restrictive lung disease. Focused respiratory examination, including auscultation and spirometry, can help predict postoperative ventilatory requirements [59]. Preoperative cardiovascular stability, including blood pressure, heart rate, and rhythm, often correlates with favorable postoperative outcomes [60]. Reduced left ventricular ejection fraction, clinical congestive heart failure, angina, active smoking, or diabetes may modestly increase the risk of delayed extubation [60]. Patients with stable, well-controlled heart failure may still be candidates if ejection fraction is adequate and symptoms are minimal.

Functional capacity, as measured by the metabolic equivalent of task (MET) score, provides insight into cardiopulmonary reserve. A MET score ≥ 4 indicates sufficient baseline fitness for most patients undergoing FTE [61]. Functional capacity may be assessed via activity questionnaires or formal cardiopulmonary exercise testing when feasible [62].

Patients with well-controlled asthma, mild COPD, or no recent exacerbations may be suitable candidates; severe COPD, home oxygen dependency, or recent respiratory infections typically necessitate prolonged postoperative ventilation [55]. Controlled hypertension, mild-to-moderate heart failure, or atrial fibrillation does not usually preclude FTE, whereas uncontrolled heart failure, significant valvular disease, or severe left ventricular dysfunction may require extended support [55]. Obesity, particularly morbid obesity, can complicate weaning due to increased work of breathing and atelectasis risk, sometimes necessitating longer positive pressure ventilation [63].

Common comorbidities such as diabetes and renal insufficiency can increase surgical risk [64]. Optimal perioperative glucose and fluid management can mitigate some risks, though severe renal impairment or poorly controlled diabetes may limit candidacy [65,66]. Risk stratification tools, including EuroSCORE and STS models, incorporate patient characteristics, comorbidities, and operative variables to guide extubation readiness [67,68].

Nutritional status impacts respiratory muscle strength and extubation success; malnourished patients may benefit from preoperative optimization [69,70]. Preoperative anxiety can also influence extubation success, and anxiolytic counseling or medication may support more effective FTE. Formal assessment of psychological readiness remains an area of ongoing investigation, with preliminary work in other surgical populations suggesting potential benefits [71,72,73].

Extubation timing is a collaborative, interdisciplinary process involving a licensed independent practitioner (LIP), nursing staff, and respiratory therapists. The LIP evaluates criteria including spontaneous tidal volumes ≥ 700 mL, maximum inspiratory pressure ≤ −40 cm H_2_O, adequate rewarming, absence of active bleeding, and sufficient cognitive status to follow commands. Coronary artery bypass graft patients are typically expected to be extubated within 24 h postoperatively. Following the extubation order, the team works collectively to wean the patient safely from mechanical ventilation.

Ultimately, selecting patients for FTE requires multidisciplinary input. Ideal candidates generally have lower surgical risk, stable comorbidities, and adequate functional capacity [9]. Careful evaluation and individualized planning can improve outcomes, reduce ICU stay, and facilitate faster postoperative recovery in patients undergoing cardiac surgery.

### 5.2. Intraoperative Implementation

Although established scoring systems, such as EuroSCORE and ANZROD, identify patients at high preoperative risk for mortality in cardiac surgery, no widely adopted scoring systems currently incorporate intraoperative variables to predict mortality or the feasibility of FTE [67,74]. Several models have been developed to predict prolonged mechanical ventilation, some externally validated, allowing risk stratification that can inform postoperative management [55,75,76,77,78]. However, relatively few studies have focused specifically on predictors of early extubation, with most research concentrating on factors associated with FTE failure or prolonged ventilation [79,80,81]. These studies primarily aim to identify modifiable intraoperative factors that anesthesiologists can address to reduce failure rates [82].

Successful FTE protocols may allow patients to bypass ICU admission, thereby offsetting healthcare costs, whereas FTE failure can increase costs due to additional ICU and post-anesthesia care unit (PACU) admissions [83,84,85]. Consequently, understanding intraoperative predictors of FTE failure is essential both clinically and economically.

Intraoperative variables consistently associated with FTE failure include CPB duration, inotrope or vasopressor requirements, and transfusion of blood products [3,84,86]. Multiple studies have demonstrated that longer CPB time predicts delayed extubation and prolonged ventilation. For instance, MacLeod et al. compared 245 FTE patients to 3007 controls and found significantly shorter CPB times in the FTE group (79 vs. 94 min, *p* < 0.0001) [86]. A predictive model developed in 3919 adults found that CPB > 120 min doubled the risk of delayed extubation, with an area under the curve of 0.782 [55]. Similarly, Hessels et al. reported that CPB > 210 min increased the likelihood of prolonged ventilation nearly fourfold [87]. Other studies have reported thresholds ranging from 77 to 130 min, with consistent associations between longer CPB and FTE failure or delayed extubation [88,89,90,91,92,93,94].

The mechanistic rationale for these associations includes CPB-induced systemic inflammatory responses due to blood contact with foreign surfaces, release of inflammatory mediators, and subsequent pulmonary edema, all of which can contribute to respiratory failure and prolonged mechanical ventilation [93,95].

In addition to CPB duration, intraoperative inotrope use has been linked to FTE failure. Retrospective analyses have shown that major inotrope use—defined as catecholamines or phosphodiesterase inhibitors for at least 30 min—predicts FTE failure (OR 5.73, *p* = 0.004) [84]. MacLeod et al. observed lower inotrope use in successfully fast-tracked patients (11.8% vs. 18.2%, *p* = 0.02) [86]. Wong et al. similarly reported post-bypass inotrope support as a risk factor for failure to extubate within 10 h of ICU admission (OR 2.28, *p* < 0.0001), with corroborating findings in other cohorts [48,91,94]. Inotrope requirement often reflects underlying ventricular dysfunction and hemodynamic instability, which may delay extubation due to concerns about patient tolerance of spontaneous breathing [96,97].

Transfusion of blood products is another intraoperative factor associated with FTE failure. London et al. identified intraoperative platelet transfusion as predictive of FTE failure (OR 10.03, *p* = 0.005), and Habib et al. reported that any blood bank transfusion significantly predicted delayed extubation (>8 h, OR 2.41, *p* < 0.0001) [84,98]. Additional studies have confirmed red blood cell and fresh frozen plasma transfusions as independent predictors of FTE failure or prolonged ventilation [87,93,99,100]. Perioperative bleeding and transfusions may reflect overall surgical complexity, prolonged CPB time, hemodilution, platelet dysfunction, and coagulation factor consumption, all of which are associated with worse outcomes [100,101].

Importantly, no single factor typically dictates extubation readiness; rather, the cumulative effect of multiple intraoperative variables determines feasibility [96]. While CPB duration, inotrope use, bleeding, and transfusion are frequently implicated, attention to hemostasis before, during, and after surgery remains a key recommendation [82]. Across the literature, intraoperative and postoperative clinical variables consistently demonstrate greater predictive value for FTE feasibility than preoperative factors [84,102].

### 5.3. Postoperative Implementation

Complete reversal of neuromuscular blockade is essential to minimize respiratory complications during extubation. Inadequate reversal can result in residual muscle weakness, increasing the risk of hypoventilation and reintubation [103]. Reversal agents such as neostigmine combined with glycopyrrolate or newer agents like sugammadex provide rapid and predictable reversal and should be selected based on the type of neuromuscular blocker used intraoperatively. Objective neuromuscular monitoring, for example, using a train-of-four ratio, is necessary to confirm full reversal prior to weaning ventilatory support [104].

Minimizing or discontinuing sedative infusions as early as feasible postoperatively facilitates neurological assessment and spontaneous breathing trials (SBT) [10]. Short-acting sedatives such as propofol and dexmedetomidine can accelerate extubation while maintaining patient comfort. Dexmedetomidine is particularly advantageous due to its sedative, anxiolytic, and analgesic effects without causing respiratory depression [53]. Regular sedation assessments using validated tools, such as the Richmond Agitation-Sedation Scale (RASS), guide titration and optimize timing for extubation [105].

Early SBTs, typically conducted within hours postoperatively, evaluate the patient’s capacity to maintain spontaneous ventilation [10]. Common approaches include low-level pressure support or T-piece trials, with close monitoring of respiratory rate, tidal volume, minute ventilation, and arterial blood gases. Hemodynamic stability is also critical. Patients should be observed carefully for signs of respiratory distress, as they remain at elevated risk of fatigue and respiratory failure shortly after extubation [106].

Although extubation was historically restricted to daytime hours to ensure access to the full clinical team, many institutions are adopting 24 h extubation protocols to optimize outcomes [3,107]. Successful implementation requires cross-departmental coordination and staff training for nurses and respiratory therapists to manage extubation safely during night shifts. Monitoring outcomes such as extubation success, reintubation rates, and complications facilitates protocol evaluation and quality improvement [108]. Implementing 24 h extubation programs, while requiring direct operational investment in 24/7 intensivist coverage and experience staff, proves cost effective [109,110]. These programs significantly reduce costly ICU days (approximately $1522 a day) and hospital charges (approximately $6000 a patient) without increasing overall hospital costs after risk adjustment [107,111,112]. FTE consistently decreases ICU stay and ventilator-associated complications, maintaining a comparable safety profile.

Adequate analgesia is crucial to prevent agitation and respiratory compromise. Multimodal analgesic strategies, often incorporating regional anesthesia (e.g., thoracic epidural or nerve blocks) alongside systemic analgesics, provide effective pain control [46]. Optimizing analgesia while reducing sedative use supports early mobilization and decreases the risk of postoperative delirium, which can complicate respiratory management [47].

Early mobilization post-extubation improves pulmonary function and reduces the incidence of atelectasis and pneumonia [51]. Mobilization may begin once patients are hemodynamically stable, progressing from passive range-of-motion exercises to sitting and ambulation under the guidance of physical therapy [52].

Overall, these postoperative strategies aim to enhance patient outcomes by reducing ICU and HLOS and optimizing resource utilization. A comprehensive, multidisciplinary approach involving anesthesiologists, surgeons, intensivists, nursing staff, and respiratory therapists is essential for successful FTE and postoperative management in cardiac surgery patients.

## 6. Discussion

FTE represents a major advancement in contemporary perioperative cardiac care, aligning closely with enhanced recovery principles aimed at improving outcomes and optimizing resource use [54]. When integrated into structured perioperative pathways that include preoperative risk stratification, tailored anesthetic techniques, and standardized postoperative management, FTE consistently demonstrates reductions in ICU length of stay, HLOS, ventilator-associated complications, and overall healthcare costs [2,4,6,60,63]. These outcomes underscore the value of coordinated care models that support timely liberation from mechanical ventilation without compromising patient safety.

The success of FTE is highly dependent on appropriate patient selection and intraoperative optimization. Factors such as functional capacity, degree of comorbidity control, airway management strategy, adequacy of neuromuscular blockade reversal, and intraoperative hemodynamic stability strongly influence readiness for early extubation and the risk of extubation failure [8,113]. These findings reinforce the importance of individualized assessment rather than rigid protocol-driven extubation timelines. Moreover, ongoing improvements in cardiopulmonary bypass management, myocardial protection, multimodal analgesia, and short-acting sedatives have broadened the population of adult cardiac surgical patients considered suitable for fast-track pathways [7].

Multidisciplinary collaboration remains a cornerstone of safe and effective implementation. Anesthesiologists, surgeons, intensivists, nurses, and respiratory therapists each play essential roles in patient assessment, protocol adherence, and rapid recognition of clinical changes after extubation [114,115,116]. Institutions adopting FTE must tailor their protocols to local patient demographics, staffing resources, and postoperative monitoring capabilities, as institutional variability directly influences extubation feasibility, safety, and outcomes [1,54].

Despite growing evidence in support of FTE, challenges persist. Particularly, variability in extubation criteria, inconsistent use of multimodal analgesia, and limited standardization across health systems pose an issue. Additionally, divergent findings across observational studies highlight the need for more rigorous synthesis of available evidence. The heterogeneity of study designs, patient populations, and perioperative practices complicates direct comparison, underscoring the need for systematic evaluation using standardized methodology.

Collectively, current evidence supports FTE as a safe, effective, and resource-conscious strategy within ERAS-based cardiac surgical care. Continued refinement of patient selection, anesthetic techniques, and postoperative pathways will be essential to maximizing the benefits of fast-track extubation while maintaining a strong safety margin for diverse adult cardiac surgical populations.

## 7. Future Directions

Future research should prioritize the development of more precise patient selection tools that incorporate function capacity assessments, frailty scoring, and real-time intraoperative physiologic markers. These approaches may help clinicians more accurately identify adult cardiac surgical patients most likely to benefit from fast-track extubation while minimizing extubation failure risk [63,113]. Additionally, greater standardization of anesthetic and analgesic strategies, particularly opioid-sparing regiments, regional anesthesia, and the use of agents such as dexmedetomidine, would reduce practice variability and strengthen protocol reproducibility across institutions.

There is also an opportunity to examine how pediatric cardiac surgery fast-track pathways, which often utilize structured extubation criteria and streamlined ICU workflows, may inform adult protocols. Pediatric programs frequently adopt early extubation as default practice, supported by enhanced multidisciplinary coordination and well-defined physiologic thresholds for readiness. These models may offer valuable insights into workflow standardization, team integration, and extubation timing that could be adapted to adult cardiac populations, particularly in high-volume or resource-variable centers. Incorporating comparative analyses or shared conceptual frameworks from pediatric fast-tracking may help refine adult protocols without altering the adult-specific evidence base.

Institutional factors also warrant further investigation, including the impact of staffing models, respiratory therapy involvement, ICU workflow efficiency, and postoperative monitoring capabilities on FTE success. Implementation-science approaches could help identify operational barriers, evaluate sustainability, and support broader adoption of standardized fast-track pathways.

Given the heterogeneity of existing studies in adult populations, a PRISMA-guided systematic review with meta-analysis remains a critical next step to more rigorously synthesize available evidence [7]. Such work would quantify outcome variability, evaluate risk profiles, and clarify which subgroups experience the greatest benefit.

Finally, integrating fast-track extubation without broader ERAS cardiac pathways, including early mobilization strategies, digital physiologic monitoring, and predictive analytics, represents a promising direction for future exploration. As hospital continue to emphasize quality improvement, cost reduction, and patient-centered perioperative care, refining both adult and cross-informative fast-track strategies will be essential to advancing recovery in modern cardiac surgery.

## Data Availability

No new data were created or analyzed in this study. Data sharing is not applicable to this article.

## Supplementary Materials

The following supporting information can be downloaded at: https://www.mdpi.com/article/10.3390/jcdd13010006/s1, Table S1: Summary of selected fast-track extubation (FTE) studies in adult cardiac surgery.

## Abbreviations

The following abbreviations are used in this manuscript:

ERAS	Enhanced recovery after surgery
HLOS	Hospital length of stay
ICU	Intensive care unit
FTE	Fast-track extubation
FTCA	Fast-track cardiac anesthesia
CABG	Coronary artery bypass graft
COPD	Chronic obstructive pulmonary disease
MET	Metabolic Equivalent of Task
LIP	Licensed Independent Practitioner
SBT	Spontaneous Breathing Trial
EuroSCORE	European System for Cardiac Operative Risk Evaluation
TAVR	Transcatheter Aortic Valve Replacement
STS	Society of Thoracic Surgeons
PACU	Post-anesthesia care unit
CPB	Cardiopulmonary bypass
ANZROD	Australian and New Zealand risk of death model
RASS	Richmond Agitation Sedation Scale
NSAID	Non-steroidal anti-inflammatory drug
