# Supplementary material for: Fast-Track Extubation After Cardiac Surgery: A Narrative Review
Source: J Cardiovasc Dev Dis. 2025 Dec 22;13(1):6. doi: 10.3390/jcdd13010006 (PMC12841624; doi:10.3390/jcdd13010006)
Supplement: Supplementary file 1 [file jcdd-13-00006-s001.zip › jcdd-3985882-supplementary.pdf]

Table S1. Summary of selected fast-track extubation (FTE) studies in adult cardiac surgery

Study (year)	Design / N	FTE definition / protocol	Reintubation rate	ICU LOS (reported)	Key predictors of FTE failure / notes
Helwani 2022	Observational pre/post; N=472	Multidisciplinary 3-hour fast-track protocol (extubation goal ≤3 h)	0.8% (post-protocol; authors report low reintubation, not increased vs pre).	No significant change in ICU LOS after implementation (median/mean reported in paper; protocol reduced ventilation time).	Longer procedure time, higher inotrope use, and patient complexity associated with delayed extubation; protocol reduced ventilation time but ICU LOS unchanged.
Cove 2016	Multidisciplinary protocol evaluation; N=201	Institutional protocol for early extubation (time-based pathway)	Reintubation rate unchanged pre vs post; low absolute rates reported.	Reduced ICU LOS post-protocol ($p<0.001$).	Protocolized approach increased rate of early extubation without increasing reintubation; successful pathways included standardized analgesia, sedation, and weaning.
Lima 2019	Narrative/systematic review of FTE programs	Summarizes multiple FTE definitions (OR extubation, ≤6–8 h, protocolized pathways)	Most studies report low reintubation rates (typically <2–5%); heterogenous.	Many studies show reduced ICU LOS with early extubation; exact values vary across cohorts.	Common predictors of failure across studies: older age, complex procedures, high inotrope/vasopressor requirement, prolonged CPB/operative time, respiratory comorbidity.
Bianchi 2021	Retrospective cohort of adult congenital heart surgery patients; N=711	"Ultra-fast-track" (very early extubation; institutionally defined)	Very low reintubation: 0.3% overall.	Shorter ICU LOS ($p<0.001$).	Selected patients (less complex, favorable physiology) were targeted; paper emphasizes patient selection for safety.
Taylor 2022	Multi-study synthesis / observational analyses	Early extubation defined as extubation within 6–8 h in many included studies	Reintubation rates low and not increased with early extubation in pooled/observational data.	Example: mean ICU stay shorter for ≤8 h extubation group (29.0 ±15.8 h vs 46.1 ±33.9 h; $p<0.001$) in one cohort.	Advanced age, high postoperative bleeding, respiratory complications, high inotrope use

					predict failure; authors stress selection.
Subramaniam 2017	Predictors study of OR extubation	OR extubation vs routine ICU extubation	Noted low reintubation in carefully selected OR-extubated patients (study focuses on predictors rather than absolute rates).	OR extubation associated with shorter postoperative stays in selected patients (specific LOS values in paper).	Predictors of success: younger age, lower surgical complexity, shorter CPB time, minimal vasoactive support.

Studies varied in; the definition of fast-track (operating room extubation, extubation within 3, 6, or 8 hours); patient selection (routine vs selected low-risk); reported outcomes (mean vs median LOS). Reintubation rates across protocolized programs are generally low (commonly <5%), but extubation failure is consistently associated with increased ICU/hospital LOS and mortality in broader critical care literature. Values in this table are taken from the cited manuscripts; differences in case-mix and outcome reporting limit direct comparison.
